# Impact of daily *Chlorella* consumption on serum lipid and carotenoid profiles in mildly hypercholesterolemic adults: a double-blinded, randomized, placebo-controlled study

**DOI:** 10.1186/1475-2891-13-57

**Published:** 2014-06-11

**Authors:** Na Hee Ryu, Yeni Lim, Ji Eeun Park, Joohee Kim, Ji Yeon Kim, Sung Won Kwon, Oran Kwon

**Affiliations:** 1 Department of Nutritional Science and Food Management, Ewha Womans University, Seoul 120-750, Republic of Korea; 2 BioFood Network, Ewha Womans University, Seoul 120-750, Republic of Korea; 3 Department of Food Science and Technology, Seoul National University of Science and Technology, Seoul 139-743, Republic of Korea; 4 CHA Bundang Medical Center, CHA University, Seongnam, Gyeonggi-do 463-712, Republic of Korea

**Keywords:** *Chlorella*, Serum lipid, Carotenoids, Human

## Abstract

**Background:**

High level of serum cholesterol is considered to be a major risk factor for cardiovascular disease (CVD). A double-blinded, randomized, placebo-controlled trial was performed to test the hypothesis that a daily intake of *Chlorella* may improve serum lipid profile through enhancement of serum carotenoid concentration in mildly hypercholesterolemic subjects.

**Methods:**

Eligible subjects (*n* = 63) were randomized to either *Chlorella* (5 g/day) or placebo for a double-blinded trial with a 2-week lead-in period and a 4-week intervention period. Serum triglycerides, total cholesterol, lipoproteins, apolipoproteins and carotenoids were assessed at the beginning and the end of the trial.

**Results:**

Compared with the control group, the *Chlorella* group exhibited remarkable changes in total cholesterol (*Chlorella −*1.6%; placebo 0.03%; *P* = 0.036), triglycerides (*Chlorella −*10.3%; placebo 11.9%; *P* = 0.002), lutein/zeaxanthin (*Chlorella* 89.6%; placebo −1.7%; *P* < 0.0001), and α-carotene (*Chlorella* 163.6%; placebo 15%; *P* < 0.0001). Improvement of serum lipids was supported by significant reductions of very low-density lipoprotein cholesterol (*Chlorella −*11%; placebo 11.8%; *P* = 0.006), apolipoprotein B (*Chlorella −*1.5%; placebo 1.7%; *P* = 0.044), non high-density lipoprotein (*Chlorella −*2.6%; placebo −0.5%; *P* = 0.032), and high-density lipoprotein/triglycerides (*Chlorella* 4.0%; placebo −9.5%; *P* = 0.023), suggesting an inhibitory effect of *Chlorella* on the intestinal absorption of dietary and endogenous lipids. Further, the changes of serum lipids appeared to be associated with the changes of serum carotenoids.

**Conclusion:**

Daily consumption of *Chlorella* supplements provided the potential of health benefits reducing serum lipid risk factors, mainly triglycerides and total cholesterol, in mildly hypercholesterolemic subjects. The effect was related to carotenoid consumption.

**Trial registration:**

WHO International Clinical Trials Registry Platform KCT0000259.

## Background

Cardiovascular disease (CVD) is one of the leading causes of death throughout the world. The World Health Organization (WHO) has predicted that the global prevalence of CVD will reach 24 million by 2030 [1]. There is increasing evidence that dietary patterns, certain foods, and food constituents have the potential to benefit CVD prevention through a variety of mechanisms. Total cholesterol (TC) and triglycerides (TG) have featured as important indicators of CVD risk for use in serum [2-5]. Recent data have demonstrated that abnormalities in the metabolism of lipoproteins and apolipoproteins are also responsible for an increased risk of CVDs, supporting the idea that they are more reliable indicator of risk than conventional serum lipid measurements [6,7].

*Chlorella* is single-celled green alga that is widely marketed as a dietary supplement or incorporated into food. Animal studies demonstrated that *Chlorella* supplementation reduced the serum cholesterol levels under high-fat or high-cholesterol diets [8-10]. Small, open-label trials yielded promising results with a focus on the improvement of serum cholesterol [11-13], but this effect has not been examined in randomized, placebo-controlled trials (RCTs) with a wider array of biomarkers. *Chlorella* is also known as a potential source of a wide spectrum of nutrients, including chlorophyll, carotenoids, minerals, vitamins, and long-chain polyunsaturated fatty acids [14]. Especially, omega-3 fatty acid and carotenoids have received considerable interests due to their roles in the prevention of chronic diseases and maintaining good health. Many investigators have demonstrated the importance of omega-3 fatty acid for reduction in serum triglyceride concentration [15,16]. The health benefits of carotenoids have been ascribed, in part, to antioxidant scavenging singlet molecular oxygen and peroxyl radicals [17]. Lutein, the most abundant carotenoid in *Chlorella*, together with zeaxanthin have been shown to be associated with eye health and function [18]. Beta-carotene are known to be inversely related to the risk of CVDs and certain cancers [19]. Alpha and β-carotene have the added advantage of being able to convert to vitamin A, which is involved in developing and preventing chronic diseases [20]. As for the relationship between carotenoids and cholesterol, most studies have been investigated with respect to the effects of the single carotenoid compound on membrane mechanics [21], intestinal absorption [22] and incorporation into liposomes [23]. To the best of our knowledge, there is no study where the carotenoid in *Chlorella* has been related to the cholesterol metabolism in humans.

We, therefore, designed a double-blinded RCT to test the hypothesis that a daily intake of *Chlorella* supplement improves serum lipid profile, which may be attributed to enhancement of serum carotenoids. To this end, we assessed serum TC, TG, lipoproteins, apolipoproteins and carotenoids (lutein/zeaxanthin, α-carotene and β-carotene) in subjects with mild hypercholesterolemia after daily intake of *Chlorella* supplement for 4 weeks. Moreover, we evaluated the relations between changes in serum lipids and changes in serum carotenoids.

## Methods

### Subjects

One hundred twenty six subjects (>20 years old) were recruited from the CHA Medical Center (Bundang, Gyeonggi-do, Korea). To be eligible, subjects were required to have a serum TC level between 5.18 and 6.48 mmol/L (mild hypercholesterolemia) at the screening visit. Exclusion criteria were: the consumption of medications and/or dietary supplements that affect lipid metabolisms; pregnancy or lactation; known hypersensitivity to the study product; the presence of familial hypercholesterolemia, CVD, hypertensions, diabetes mellitus, hepatic diseases, kidney diseases, thyroid diseases or other systemic diseases. The study protocol was approved by the Institutional Review Boards of CHA Medical Center and Ewha Womans University (Seoul, Korea).

### Test samples

Tablets composed of *Chlorella* (*Chlorella vulgaris*) powder or lactose powder (color-matched placebo) provided by Daesang Corp. (Seoul, Korea) were used in this trial. The *Chlorella* tablet (650 mg) contained 416 mg of *Chlorella* powder with the following nutritional composition (per 100 g): 5.4 g moisture, 60.6 g protein, 3.7 g total carbohydrates, 12.8 g total lipids, 13.0 g dietary fiber, 4.5 g ash, 58,900 IU vitamin A, 74 mg vitamin C, 22.8 mg vitamin E, and 2.4 g chlorophylls [10]. The carotenoid content of *Chlorella* powder was determined in our laboratory by using a high-performance liquid chromatography (HPLC) method [24] and the results are as follows (per 100 g): 260 mg lutein, 5 mg zeaxanthin, 24 mg α-carotene, and 17 mg β-carotene.

### Study design

After initial screening and the following 2-week lead-in phase, 68 eligible subjects (44 female and 19 male) were randomized into either *Chlorella* or placebo in a double-blinded fashion. The participants were advised to maintain their usual level of activity and diet, but to avoid certain high-cholesterol and high-carotenoid foods during the lead-in and the intervention days. They were also advised to take 4 tablets of test sample with water immediately after each meal, a total of 12 tablets (5 g as *Chlorella* powder) a day, for 4 weeks. To monitor the subjects’ dietary compliances and assess their nutrient intake, three-day food diaries (including two weekdays and one weekend day) were completed at the beginning and end of the study. Energy and nutrient intakes were calculated using a computer-aided nutritional analysis program (CAN-pro 3.0, Korean Nutrition Society, Seoul, Korea).

### Measurements

Following completion of the lead-in phase and the 4-week intervention period, overnight fasting venous blood was collected from a vein on the flexor side of the arm into a serum separator tubes (BD Biosciences, San Jose, CA, USA). The concentrations of TC, TG, low-density lipoprotein-cholesterol (LDL-C), and high-density lipoprotein-cholesterol (HDL-C) were simultaneously measured using an automatic analyzer (Hitachi 7600, Hitachi High-Technologies Co., Osaka, Japan). Very low-density lipoprotein-cholesterol (VLDL-C) and non-HDL-C were calculated as TG/2.2 and the difference between TC and HDL-C, respectively [25]. Serum apolipoproteins (apo A1, apo B and apo E) were measured using a BNII nephelometer (Siemens, Munich, Germany). Serum carotenoids were analyzed after deproteinizing with ethanol containing the internal standard (α-tocopherol acetate) and then extracting with hexane. After evaporation, the residue was dissolved in mobile phase and quantified by using HPLC (Shiseido SP 3023) [24].

### Statistics

We estimated the sample size needed to detect between group differences on serum TC at the end of 4-week intervention period to be 34 subjects per group. With these estimates and a 2-sided α = 0.05, the study had greater than 80% power with up to 10% dropout. The statistical analyses were performed with the SAS program package, version 9.3 (SAS Institute, Cary, NC, USA). The normal distribution of each variable was tested before statistical testing and logarithmic transformation was performed on skewed variables. Baseline comparisons between groups were determined using Student’s *t*-test for independent samples. Differences in means for the efficacy of *Chlorella* versus placebo were analyzed by a one-way analysis of covariance (ANCOVA) after adjusting for the baseline values as covariates. Differences in means for absolute change from baseline to 4-week within groups were evaluated using paired *t*-tests. Pearson’s correlation was used to determine the strength of the relationships between the serum lipids and the serum carotenoids for all participants, irrespective of treatment group. For descriptive purposes, mean values of untransformed and unadjusted variables are presented. A two-tailed value of *P* < 0.05 was considered statistically significant.

## Results

### Subject characteristics and diet monitoring

**Figure 1:**
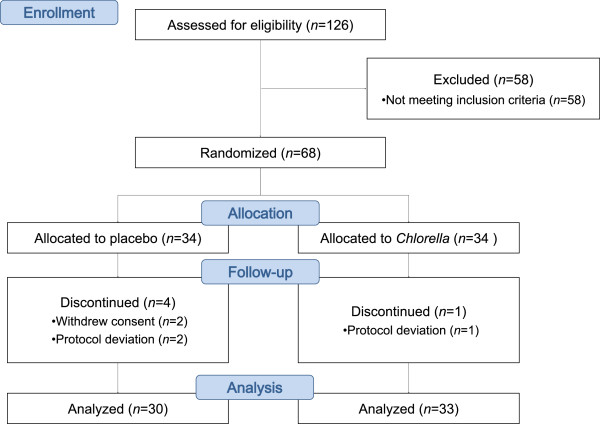
CONSORT flow diagram.

Of the 68 participants who commenced the study, 63 completed the endpoint assessments (*Chlorella*, *n* = 33; placebo, *n* = 30). Five participants (4 in the placebo group and 1 in the *Chlorella* group) withdrew during the intervention due to either protocol deviations (*n* = 3) or consent withdrawals (*n* = 2) (Figure 1). No serious adverse events or side effects were observed during the intervention period.

The participants represented a mildly hypercholesterolemic population with a mean level of 5.64 ± 0.07 mmol/L TC, 3.26 ± 0.07 mmol/L LDL-C, and 1.43 ± 0.05 mmol/L HDL-C. The randomization was successful; the two groups did not differ significantly at baseline in terms of the measured endpoints (Table 1). Furthermore, the data from the dietary records indicated that the participants generally consumed diets with similar levels of energy intake, carbohydrates, proteins, fats and cholesterol at baseline and during the study. Carotenoid intake increased significantly by supplementing 5 g of *Chlorella* as compared with placebo (*P* = 0.0003) (Table 2).

**Table 1:** Baseline characteristics of subjects who completed the intervention trial

**Variables**	**Control (*n*** **= 30)**	***Chlorella *** **(*n*** **= 33)**	***P*-value**
Age	50.9 ± 1.6	48.2 ± 1.4	0.281
Female/male (n)	22/8	22/11	0.565
BMI (kg/m^2^)	24.5 ± 0.5	23.9 ± 0.6	0.488
Fasting glucose (mmol/L)^#^	5.5 ± 0.1	5.6 ± 0.1	0.499
Systolic BP (mmHg)	120.1 ± 2.1	120.0 ± 2.0	0.981
Diastolic BP (mmHg)	75.8 ± 1.3	76.4 ± 1.7	0.787
Lipid profiles			
TC (mmol/L)	5.68 ± 0.10	5.60 ± 0.10	0.587
TG (mmol/L)^#^	1.49 ± 0.14	1.90 ± 0.23	0.137
LDL-C (mmol/L)	3.34 ± 0.10	3.18 ± 0.09	0.262
HDL-C (mmol/L)	1.40 ± 0.06	1.43 ± 0.06	0.914
VLDL-C (mmol/L)^#^	0.68 ± 0.06	0.86 ± 0.11	0.163
non-HDL-C (mmol/L)	4.26 ± 0.11	4.17 ± 0.11	0.568
Apo A1 (g/L)	1.47 ± 0.05	1.52 ± 0.05	0.493
Apo B (g/L)	1.07 ± 0.03	1.08 ± 0.02	0.886
Apo E (g/L)^#^	0.05 ± 0.00	0.06 ± 0.00	0.747

All values are means ± SEM. Student’s *t*-test or chi-square test was used to analyze the data. *BMI* body mass index; *BP* blood pressure; *TC* total cholesterol; *TG* triglycerides; *LDL-C* low-density lipoprotein cholesterol; *HDL-C* high-density lipoprotein cholesterol; *VLDL-C* very low-density lipoprotein cholesterol; *Apo* apo-lipoprotein.

^#^Tested after log transformation.

**Table 2:** Mean daily intake of energy and selected nutrients at baseline and after intervention

**Nutrients**	**Placebo (*n*** **= 30)**		***Chlorella *** **(*n*** **= 33)**		***P*-value**^**§**^	
	**Week 0**	**Week 4**	**Week 0**	**Week 4**	**Week 0**	**Week 4**
Energy (kcal)	1527 ± 71	1560 ± 66	1695 ± 85	1664 ± 66	0.135	0.270
Carbohydrate (g)	234.8 ± 10.0	240.2 ± 8.0	250.7 ± 11.5	247.1 ± 9.7	0.304	0.591
Dietary fibers (g)	19.9 ± 1.0	22.6 ± 1.1*	19.7 ± 1.1	21.6 ± 1.2*	0.900	0.539
Protein (g)	61.5 ± 3.1	62.5 ± 3.2	69.1 ± 4.2	67.5 ± 3.1	0.183	0.280
Total fat (g)	38.8 ± 2.5	38.9 ± 2.7	45.9 ± 3.4	45.4 ± 2.8	0.114	0.108
SFA (g)	5.7 ± 0.5	6.0 ± 0.8	7.2 ± 0.9	6.4 ± 0.6	0.346	0.480
MUFA (g)	7.1 ± 0.6	7.3 ± 0.9	8.4 ± 1.0	8.2 ± 0.7	0.457	0.273
PUFA (g)	6.1 ± 0.6	6.4 ± 0.6	7.0 ± 0.6	6.2 ± 0.4	0.260	0.854
Cholesterol (mg)	271.9 ± 26.6	289.3 ± 27.8	303.2 ± 27.7	269.2 ± 21.3	0.339	0.564
β-carotene (mg)^#^	2.8 ± 0.2	3.1 ± 0.3	3.0 ± 0.4	4.7 ± 0.4*	0.711	0.0003

All values are means ± SEM. Intakes were estimated from three-day food records by using CAN-pro (Korean Nutrition Society, Seoul, Korea). Intakes of test products were included in the analysis. *SFA* saturated fatty acids; *MUFA* monounsaturated fatty acids; *PUFA* polyunsaturated fatty acids.

^#^Tested after log transformation.

*Significantly different within a group, *P* < 0.05 (paired *t*-test).

^§^Comparison between the placebo and the *Chlorella* group at week 0 and week 4, respectively (Student’s *t*-test).

### Serum lipid and carotenoid profiles

**Figure 2:**
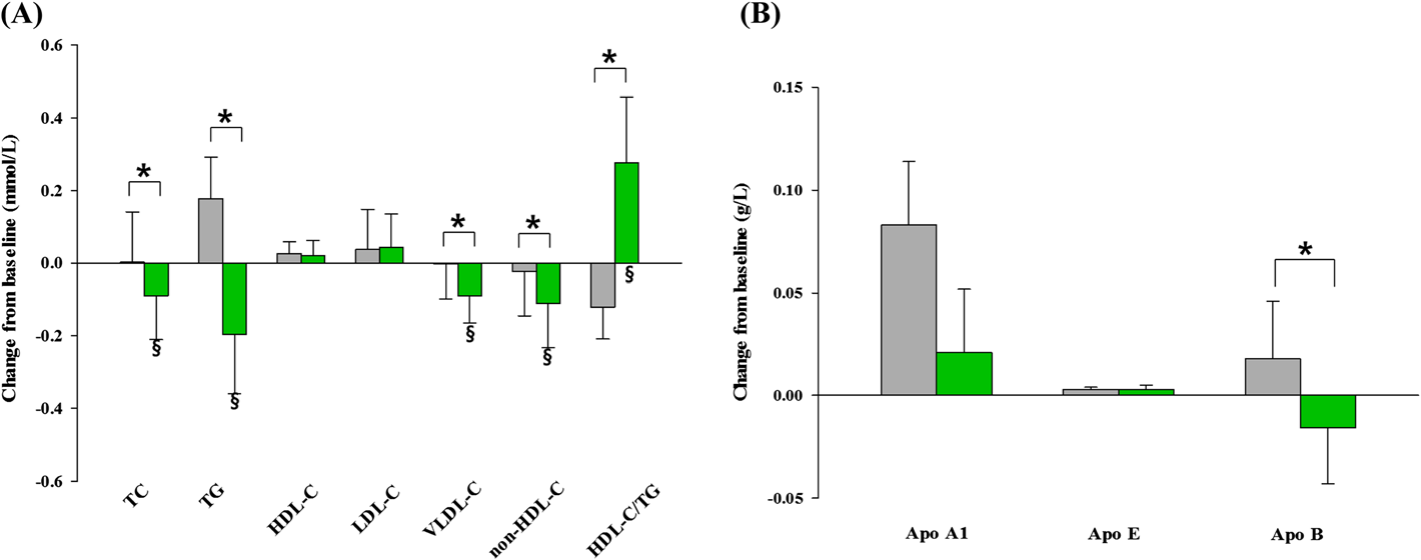
**Mean changes of serum lipids profile in subjects with mild hypercholesterolemia.** Panel **A** shows the changes of lipids and lipoproteins for *Chlorella* (green rectangle, *n* = 33) and placebo (gray rectangle, *n* = 30). Panel **B** shows the changes of apolipoproteins. The data represent means ± SEM. *P < 0.05 compared between groups using ANCOVA after adjusting for the baseline value. ^§^*P* < 0.05 compared to baseline using paired *t*-test.

While no significant changes were observed in the placebo group, the *Chlorella* group showed significant reductions from baseline in TC (1.6% reduction, *P* = 0.046), TG (10.3% reduction, *P* = 0.029), VLDL-C (11% reduction, *P* = 0.029), non-HDL-C (2.6% reduction, *P* = 0.024), and HDL-C/TG (4.0% increase. *P* = 0.035). The differences in means between groups were also significant for TC (*P* = 0.036), TG (*P* = 0.002), VLDL-C (*P* = 0.032), non-HDL-C (*P* = 0.006), and HDL-C/TG (*P* = 0.023), indicating true supplement effects (Figure 2A). The serum LDL-C and HDL-C levels, however, did not change significantly in the Chlorella group during the study; thus, there were no significant differences between the groups. Apolipoproteins were also measured, showing a significant decrease of apo B concentration in the *Chlorella* group (*P* = 0.129) and increased in the control group (*P* = 0.113), resulting in a significant difference between the two groups (*P* = 0.044). However, there were no significant differences in apo A1 and apo E between the two groups (Figure 2B).

**Figure 3:**
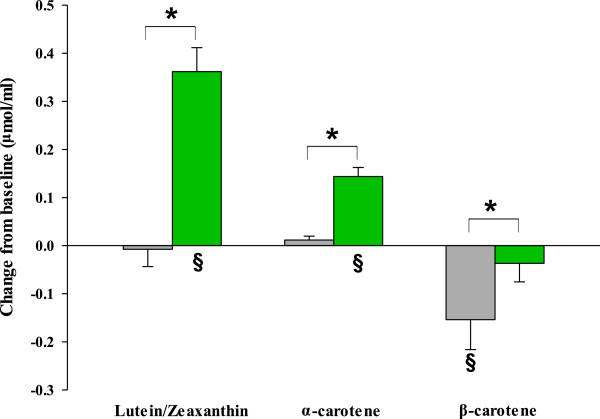
**Mean changes of serum carotenoids profile in subjects with mild hypercholesterolemia.***Chlorella* (green rectangle, *n* = 33) and placebo (gray rectangle, *n* = 30). The data represent means ± SEM. *P < 0.05 compared between groups using ANCOVA after adjusting for the baseline value. ^§^*P* < 0.05 compared to baseline using paired *t*-test.

The changes in serum carotenoids were shown in Figure 3. As predicted based on the carotenoid composition of *Chlorella*, the serum lutein/zeaxanthin and α-carotene levels were significantly increased followed by *Chlorella* supplementation within groups (*P* < 0.0001) and compared with the control group (*P* < 0.0001). Unexpectedly serum β-carotene concentrations exhibited a significant decrease from baseline value in the placebo group compared with the *Chlorella* group (*P* = 0.043), resulting in a significant difference between the two groups (*P* = 0.029).

### Correlation between changes in serum lipids and carotenoids

**Figure 4:**
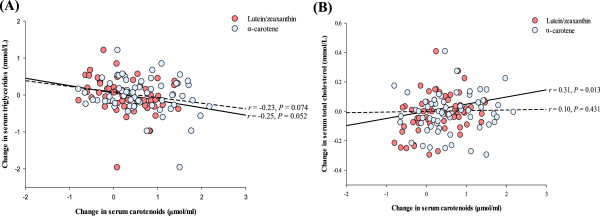
**Correlation between the changes of serum carotenoids and lipids.** Panel **A** shows a correlation between lutein/zeaxanthin or α-carotene and TG for all subjects (*n* = 63). Panel **B** shows the same analysis for TC. The Pearson correlation *r*- and *P*-values for each curve are shown adjacent to the line in the respective panel.

The analysis of correlation revealed that the serum TG concentrations showed a tendency toward a negative correlation with the serum lutein/zeaxanthin (*r* = −0.25, *P* = 0.052) and α-carotene (*r* = −0.23, *P* = 0.074) for all groups (Figure 4A). However, a contrary effect was found in the relationship between the serum TC and the serum lutein/zeaxanthin (*r* = 0.31, *P* = 0.013). No correlation was found between the serum TC and the serum α-carotene (Figure 4B).

## Discussion

To the best of our knowledge, the present study is the first evidence offered to support the hypothesis that the regular consumptions of *Chlorella* supplement (5 g/day) over 4 weeks significantly reduced serum TG, TC, non-HDL-C, VLDL-C, HDL-C/TG, and apo B in subjects with mild hypercholesterolemia using RCT study design. This study also demonstrated that daily consumption of Chlorella supplement resulted in significant increases inserum lutein/zeaxanthin and α-carotene concentrations. In addition, the changes of serum TG and TC appeared to be associated with the changes in serum lutein/zeaxanthin and α-carotene.

Some studies in open-label (“before and after”) design with relatively small numbers of subjects have shown the effect of *Chlorella,* focusing on the serum cholesterol levels rather than overall lipid and carotenoid profiles. Okuda *et al.*[11] showed that the daily intakes of 5 g of *Chlorella* for three months significantly lowered the TC of subjects with hypercholesterolemia (*n* = 16) to almost the normal level. In another study, Youko *et al.*[12] demonstrated reductions in TC and LDL-C concentrations after 9 g of *Chlorella* supplementation for 12 months in patients with hyperlipidemia (*n* = 9). Sansawa *et al.*[13] found that the daily ingestion of 3 g of *Chlorella* for three months lowered serum TC and LDL-C in patients with mild hypercholesterolemia (*n* = 20). These small studies suggested a potential of *Chlorella* supplementation for reductions of TC at doses of 3–9 g, providing a rationale for further evaluations of *Chlorella* in a randomized, placebo-controlled trials.

In the lipoprotein lipolytic cascade, apo B is required for the secretion of VLDL from the liver and converted to intermediate-density lipoprotein (IDL) by lipoprotein lipase in the endothelial surface of capillaries. The IDL particles can be rapidly taken up and then catabolized mainly in the liver *via* apo E, a high-affinity ligand for the receptor. However, some IDL particles escape hepatic uptake and remain in the circulation due to lack of apo E and are further converted to LDL [26]. The binding affinity of apo B was relatively low, and thus, LDL particles circulated for a relatively prolonged period before binding to LDL receptors throughout the body [27,28]. There is a systematically only one apo B per LDL particle and thus plasma level of apo B is strongly associated with LDL particle number, which is an important predictor of cardiovascular risks [29]. Conversely, the HDL accepts cholesterol from peripheral cells and transports it to the liver for bile production. Apo A1 is the major protein constituent of HDL particles, mediating reverse cholesterol transport [30]. It is important to note that the most pronounced effect of *Chlorella* in the current study were decreases in VLDL and apo B concentrations. This result implicates that there are slowed secretion of apo B and VLDL into the circulation or faster catabolism of VLDL in the liver. This is speculative but the first part is consistent with the previous findings that *Chlorella* plays an important role in inhibiting the intestinal absorptions of dietary lipids in animals [8-10]. In addition, lowering apo B compromises lowering LDL particle number and promotes CVD risk. It is also worth noting that, in the present study, four weeks of *Chlorella* supplementation produced no noticeable effects on HDL-C, apo E, and apo A1 compared with the control group, implicating that reverse cholesterol transport was unaffected by *Chlorella* consumption. The design of the current study did not allow the direct determination of cholesterol absorption. Considering all the results together, however, we can assume that the inhibition of intestinal lipid absorption may have caused the serum lipid profile changes observed in the *Chlorella* group.

*Chlorella* provides a wide range of nutrients and phytochemicals that work synergistically to optimize lipid metabolism. It contains a relatively high percentage of omega-3 fatty acids [14], which are known not to inhibit chylomicron assembly in the intestine, but to inhibit VLDL assembly in the liver [15]. *Chlorella* is a good source of dietary fiber that affects lymphatic cholesterol and triglyceride absorption by increasing gut viscosity, altering the composition of the bile acid pool, or producing fermentation products in the intestine [31]. *Chlorella* is also a good source of carotenoids and it significantly increased serum concentration of lutein/zeaxanthin and α-carotene. The steps of carotenoid absorption are similar to those for dietary lipids: the release from the food matrix, solubilization in mixed micelles, packaging into chylomicrons, and secretion into the lymphatic system [32]. Therefore, one can assume that the presence of increased concentrations of carotenoids released from *Chlorella* compete with dietary lipids for incorporation and transport in lipoproteins, therefore causing the decreased serum lipid levels. In a previous animal study Nicolle *et al.*[33] investigated the effects of carrot supplementation on lipid metabolism in rats, suggesting that dietary carotenoid consumption modifies lipid absorptions. In this study, we found that individuals with higher levels of serum lutein/zeaxanthin and α-carotene had greater potentials to reduce serum TG concentrations, supporting the notion that carotenoids may inhibit TG absorption. However, contrary to TG, no or weak positive correlation was found between the changes in serum carotenoids and the changes in TC. One possible explanation for these conflict may be that the polar carotenoids are mostly distributed in association with cholesterol-rich lipoproteins in the serum [32], which masks the degree of inhibitory effects of carotenoids on cholesterol absorption. This notion is further supported by the results of Loane *et al.*[34], who reported a positive association between serum lutein concentration and both serum LDL and HDL level among healthy subjects.

There are several limitations in this study. First, the suggested molecular mechanism by which *Chlorella* are thought to exert their effect was not directly determined. Despite this limitation, however, our findings raised the possibility that the effects of *Chlorella* on the inhibition of intestinal absorption of both dietary and endogenous lipids may have caused the serum lipid profile changes. Another limitation of this study is that there was no more biochemical data to show the localization of carotenoids in lipoproteins. Future studies are warranted to prove these initial findings.

## Conclusions

The present study revealed that *Chlorella* appears to have beneficial health effects on the serum lipid profiles of mildly hypercholesterolemic subjects at least in part via improvement of serum carotenoid profiles. Our findings also suggest that possible effects of *Chlorella* on serum lipids might be caused by a suppression of intestinal lipid absorption attributable to increased levels of highly polar carotenoids from *Chlorella*. Although further studies are required to determine the direct effects of *Chlorella* for the absorptions of dietary lipids, the results of this study provide evidence to previous observations, which suggest that *Chlorella* should be included on the list for recommending heart-healthy dietary supplements.

## Abbreviations

CVD: Cardiovascular disease; TC: Total cholesterol; TG: Triglycerides; RCT: Randomized-placebo-controlled trial; VLDL: Very low-density lipoprotein; LDL: Low-density lipoprotein; HDL: High-density lipoprotein.

## Competing interests

The authors have no conflict of interest.

## Authors’ contribution

OK, SWK, JYK and JK were responsible for conception and study design; OK and SWK were responsible for the supervision of the human study, sample handling, conduction of the analyses, and statistical analysis; NHR and JEP conducted the study and performed analyses of the data; YL and JK performed the statistical analysis; YL and OK prepared the manuscript; all authors read and approved the final manuscript.

## References

[B1] Lloyd-Jones D Adams R Carnethon M De Simone G Ferguson TB Flegal K Ford E Furie K Go A Greenlund K Hasse N Hailpern S Ho M Howard V Kissela B Kittner S Lackland D Lisabeth L Marelli A McDermott M Meigs J Mozaffarian D Nichol G O'Donnell C Roger V Rosamond W Sacco R Sorlie P Stafford R Steinberger J Heart disease and stroke statistics–2009 update: a report from the American Heart Association Statistics Committee and Stroke Statistics Subcommittee Circulation 2009 119 e21 e181 19075105 10.1161/CIRCULATIONAHA.108.191261

[B2] McPherson R Frohlich J Fodor G Genest J Canadian Cardiovascular Society Canadian Cardiovascular Society position statement–recommendations for the diagnosis and treatment of dyslipidemia and prevention of cardiovascular disease Can J Cardiol 2006 22 913 927 10.1016/S0828-282X(06)70310-5 16971976 PMC2570238

[B3] Mora S Otvos JD Rifai N Rosenson RS Buring JE Ridker PM Lipoprotein particle profiles by nuclear magnetic resonance compared with standard lipids and apolipoproteins in predicting incident cardiovascular disease in women Circulation 2009 119 931 939 10.1161/CIRCULATIONAHA.108.816181 19204302 PMC2663974

[B4] Nordestgaard BG Benn M Schnohr P Tybjaerg-Hansen A Nonfasting triglycerides and risk of myocardial infarction, ischemic heart disease, and death in men and women JAMA 2007 298 299 308 10.1001/jama.298.3.299 17635890

[B5] Sarwar N Sandhu MS Ricketts SL Butterworth AS Di Angelantonio E Boekholdt SM Ouwehand W Watkins H Samani NJ Saleheen D Lawlor D Reilly MP Hingorani AD Talmud PJ Danesh J Triglyceride-mediated pathways and coronary disease: collaborative analysis of 101 studies Lancet 2010 375 1634 1639 20452521 10.1016/S0140-6736(10)60545-4 PMC2867029

[B6] Sacks FM Alaupovic P Moye LA Cole TG Sussex B Stampfer MJ Pfeffer MA Braunwald E VLDL, apolipoproteins B, CIII, and E, and risk of recurrent coronary events in the Cholesterol and Recurrent Events (CARE) trial Circulation 2000 102 1886 1892 10.1161/01.CIR.102.16.1886 11034934

[B7] Contois JH McConnell JP Sethi AA Csako G Devaraj S Hoefner DM Warnick GR Practices ALaVDDWGoB Apolipoprotein B and cardiovascular disease risk: position statement from the AACC lipoproteins and vascular diseases division working group on best practices Clin Chem 2009 55 407 419 10.1373/clinchem.2008.118356 19168552

[B8] Sano T Tanaka Y Effect of dried, powdered Chlorella vulgaris on experimental atherosclerosis and alimentary hypercholesterolemia in cholesterol-fed rabbits Artery 1987 14 76 84 3566534

[B9] Shibata S Oda K Onodera-Masuoka N Matsubara S Kikuchi-Hayakawa H Ishikawa F Iwabuchi A Sansawa H Hypocholesterolemic effect of indigestible fraction of Chlorella regularis in cholesterol-fed rats J Nutr Sci Vitaminol (Tokyo) 2001 47 373 377 10.3177/jnsv.47.373 11922110

[B10] Lee HS Park HJ Kim MK Effect of Chlorella vulgaris on lipid metabolism in Wistar rats fed high fat diet Nutr Res Pract 2008 2 204 210 10.4162/nrp.2008.2.4.204 20016720 PMC2788184

[B11] Masso O Hasegawa T Sonoda M Okabe T Tanaka Y The effects of Chlorella on the levels of cholesterol in serum and liver Jap J Nutr 1975 33 8

[B12] Fujiwara Y Hirakawa K Kunihiro S Effect of Long-term administration of chlorella tablets on hyperlipemia J Jpn Soc Nutr F Sci 1990 43 167 173 10.4327/jsnfs.43.167

[B13] Sansawa H Inoue K Shirai T Effect of Chlorella tablet ingestion on mild hypercholesterolemic patients Nippon Shokuhin Kagaku Kaishi 2002 49 6

[B14] Bocanegra A Bastida S Benedí J Ródenas S Sánchez-Muniz FJ Characteristics and nutritional and cardiovascular-health properties of seaweeds J Med Food 2009 12 236 258 10.1089/jmf.2008.0151 19459725

[B15] Park Y Harris WS Omega-3 fatty acid supplementation accelerates chylomicron triglyceride clearance J Lipid Res 2003 44 455 463 10.1194/jlr.M200282-JLR200 12562865

[B16] Connor WE Importance of n-3 fatty acids in health and disease Am J Clin Nutr 2000 71 171S 175S 10617967 10.1093/ajcn/71.1.171S

[B17] Christaki E Florou-Paneri P Bonos E Microalgae: a novel ingredient in nutrition Int J Food Sci Nutr 2011 62 794 799 10.3109/09637486.2011.582460 21574818

[B18] Richer S Stiles W Statkute L Pulido J Frankowski J Rudy D Pei K Tsipursky M Nyland J Double-masked, placebo-controlled, randomized trial of lutein and antioxidant supplementation in the intervention of atrophic age-related macular degeneration: the Veterans LAST study (Lutein Antioxidant Supplementation Trial) Optometry 2004 75 216 230 10.1016/S1529-1839(04)70049-4 15117055

[B19] Johnson EJ The role of carotenoids in human health Nutr Clin Care 2002 5 56 65 10.1046/j.1523-5408.2002.00004.x 12134711

[B20] Rao AV Rao LG Carotenoids and human health Pharmacol Res 2007 55 207 216 10.1016/j.phrs.2007.01.012 17349800

[B21] Socaciu C Bojarski P Aberle L Diehl H Different ways to insert carotenoids into liposomes affect structure and dynamics of the bilayer differently Biophys Chem 2002 99 1 15 10.1016/S0301-4622(02)00111-4 12223235

[B22] During A Dawson HD Harrison EH Carotenoid transport is decreased and expression of the lipid transporters SR-BI, NPC1L1, and ABCA1 is downregulated in Caco-2 cells treated with ezetimibe J Nutr 2005 135 2305 2312 16177187 10.1093/jn/135.10.2305

[B23] Socaciu C Jessel R Diehl HA Competitive carotenoid and cholesterol incorporation into liposomes: effects on membrane phase transition, fluidity, polarity and anisotropy Chem Phys Lipids 2000 106 79 88 10.1016/S0009-3084(00)00135-3 10878237

[B24] Zonta F Stancher B Marletta GP Simultaneous high-performance liquid chromatographic analysis of free carotenoids and carotenoid esters J Chromatogr 1987 403 207 215 3680410 10.1016/s0021-9673(00)96354-4

[B25] Friedewald WT Levy RI Fredrickson DS Estimation of the concentration of low-density lipoprotein cholesterol in plasma, without use of the preparative ultracentrifuge Clin Chem 1972 18 499 502 4337382

[B26] Kita T Brown MS Bilheimer DW Goldstein JL Delayed clearance of very low density and intermediate density lipoproteins with enhanced conversion to low density lipoprotein in WHHL rabbits Proc Natl Acad Sci U S A 1982 79 5693 5697 10.1073/pnas.79.18.5693 6957885 PMC346971

[B27] Packard CJ Demant T Stewart JP Bedford D Caslake MJ Schwertfeger G Bedynek A Shepherd J Seidel D Apolipoprotein B metabolism and the distribution of VLDL and LDL subfractions J Lipid Res 2000 41 305 318 10681415

[B28] Ishibashi S Brown MS Goldstein JL Gerard RD Hammer RE Herz J Hypercholesterolemia in low density lipoprotein receptor knockout mice and its reversal by adenovirus-mediated gene delivery J Clin Invest 1993 92 883 893 10.1172/JCI116663 8349823 PMC294927

[B29] Lamarche B Tchernof A Moorjani S Cantin B Dagenais GR Lupien PJ Després JP Small, dense low-density lipoprotein particles as a predictor of the risk of ischemic heart disease in men. Prospective results from the Québec Cardiovascular Study Circulation 1997 95 69 75 10.1161/01.CIR.95.1.69 8994419

[B30] Borecki IB Laskarzewski P Rao DC Genetic factors influencing apolipoprotein AI and AII levels in a kindred with premature coronary heart disease Genet Epidemiol 1988 5 393 406 10.1002/gepi.1370050604 3145239

[B31] Queenan KM Stewart ML Smith KN Thomas W Fulcher RG Slavin JL Concentrated oat beta-glucan, a fermentable fiber, lowers serum cholesterol in hypercholesterolemic adults in a randomized controlled trial Nutr J 2007 6 6 10.1186/1475-2891-6-6 17386092 PMC1847683

[B32] Erdman JW Bierer TL Gugger ET Absorption and transport of carotenoids Ann N Y Acad Sci 1993 691 76 85 10.1111/j.1749-6632.1993.tb26159.x 8129321

[B33] Nicolle C Cardinault N Aprikian O Busserolles J Grolier P Rock E Demigné C Mazur A Scalbert A Amouroux P Rémésy C Effect of carrot intake on cholesterol metabolism and on antioxidant status in cholesterol-fed rat Eur J Nutr 2003 42 254 261 10.1007/s00394-003-0419-1 14569406

[B34] Loane E Nolan JM Beatty S The respective relationships between lipoprotein profile, macular pigment optical density, and serum concentrations of lutein and zeaxanthin Invest Ophthalmol Vis Sci 2010 51 5897 5905 10.1167/iovs.09-4878 20574027

